# Anterior Penetrating Neck Injury: A Path to the Cervical Spine

**DOI:** 10.7759/cureus.35370

**Published:** 2023-02-23

**Authors:** Duarte Gil Alves, Jessica Sousa, Vítor Ferreira, João Almeida Pinto, Nuno Teixeira

**Affiliations:** 1 General Surgery, Hospital Dr. Nélio Mendonça, Funchal, PRT; 2 Radiology, Centro Hospitalar Universitario do Porto EPE, Porto, PRT; 3 Vascular Surgery, Centro Hospitalar do Tâmega e Sousa, Penafiel, PRT; 4 General Surgery, Centro Hospitalar do Tâmega e Sousa, Penafiel, PRT

**Keywords:** steel blade, ct (computed tomography) imaging, acute care surgery and trauma, neck trauma, carotid artery surgery, knife injury, penetrating stab wound, penetrating trauma neck

## Abstract

While rare in incidence, penetrating neck injuries are often life-threatening. When a patient’s physiological status is appropriate, the first step in treatment should be a detailed preoperative imaging assessment. Formulating a treatment plan that includes computed tomography (CT) imaging and discussing the surgical approach with a multidisciplinary team before operating allows for a successful selective surgical approach.

The authors report the case of a Zone II penetrating injury with a right laterocervical entry wound in which an impaled blade with an inferomedial oblique path pierced deeply into the cervical spine. The blade missed multiple vital structures in the neck, such as the common carotid artery, jugular vein, trachea, and esophagus. The patient underwent a formal neck exploration, and controlled extraction of the blade under direct vision was achieved. Therefore, the author's recommendation for implementing any management algorithm for penetrating neck injuries should rely primarily on a multidisciplinary selective approach.

## Introduction

The neck is a challenging anatomical region, especially in traumatic injuries. Multiple structures from the vascular, respiratory, neurological, and digestive systems pass through this area, which features high density and relatively low protection. Penetrating neck injuries - those that breach the platysma - are therefore often associated with serious injury or death [1]. Since the carotid artery is often involved, exsanguination is the most frequent cause of death [2].

No international consensus guidelines exist regarding the management of penetrating neck injuries; consequently, clinicians must rely on management algorithms from high-volume trauma centers such as those in South Africa [3,4]. In this article, the authors aim to raise emergency physicians’ awareness of penetrating neck injury management principles. Although neck injuries comprise only 5%-10% of all trauma cases, their high mortality rates demand a precise, standardized approach [5,6].

## Case presentation

An 85-year-old female with no relevant past medical history was a burglary victim. The patient recounted that as she watched television, the attacker approached from behind and stabbed her in the right side of her neck. Fully conscious, she called for prehospital emergency care services and was transported to a major hospital center.

Upon emergency department admission, the patient presented with pain on the right side of the neck as well as discomfort associated with neck movement. She was calm and alert. At the primary survey, the patient’s airway was patent with ventilation intact; her peripheral oxygen saturation was 96% on room air, and neither subcutaneous emphysema nor tracheal deviation was identified. The patient’s blood pressure measured 119/50 mmHg with a heart rate of 67 beats per minute, capillary refill time of < 2 seconds, and no signs of active bleeding.

**Figure 1:**
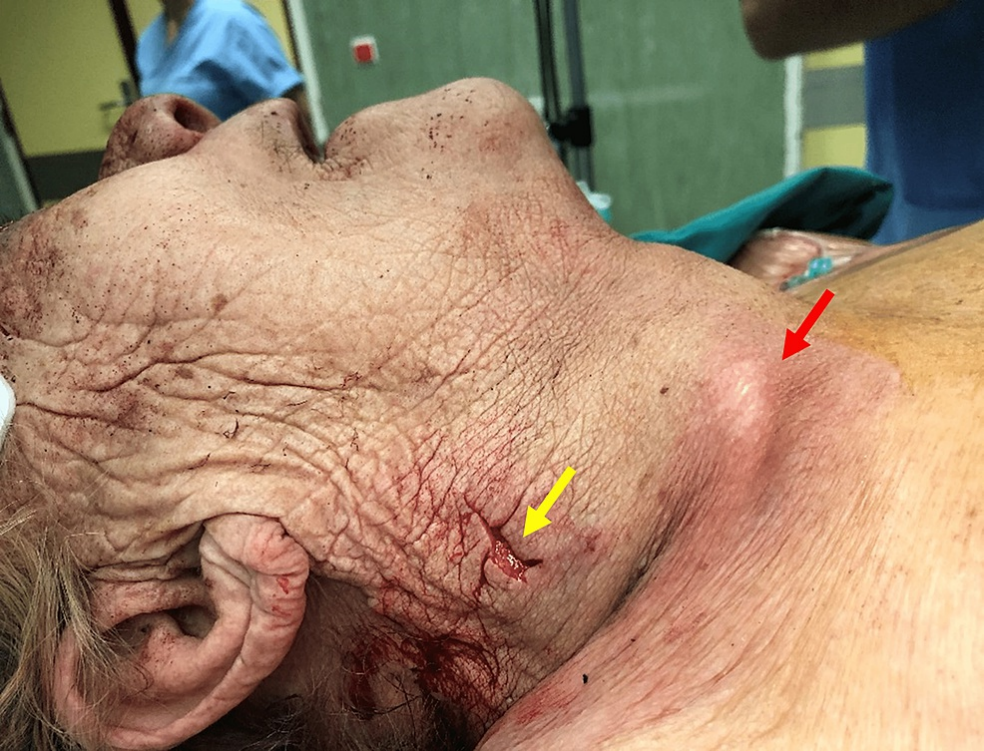
Right side view of the patient's neck during physical examination Side view of a 1.5 cm horizontal stab wound on the medial third of the right sternocleidomastoid muscle (yellow arrow). A skin prominence, consistent with the blade’s tang, was identified approximately 3 cm inferior to the suspected entry wound (red arrow).

During the physical examination, the patient was found to have a 1.5 cm horizontal stab wound on the medial third of the right sternocleidomastoid muscle that appeared to breach the platysma muscle. Approximately 3 cm inferior to the suspected entry wound, one could notice an unknown skin prominence that was consistent at palpation (Figure 1).

Investigations

**Figure 2:**
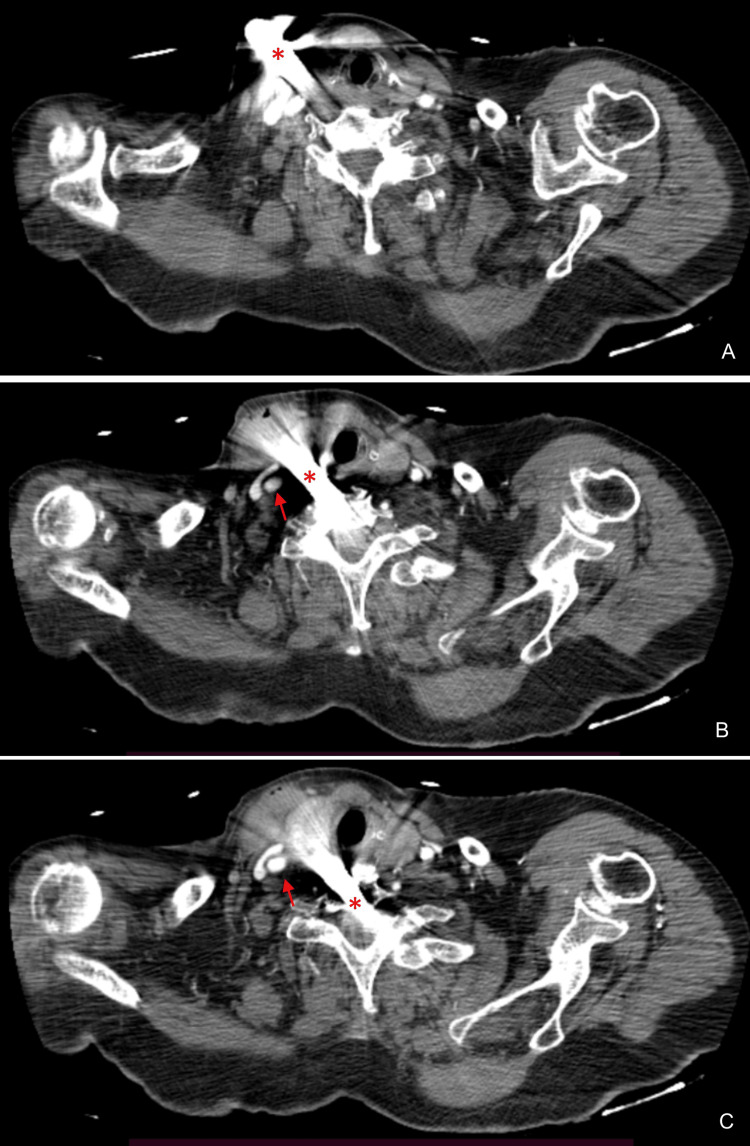
Axial enhanced computed tomography (CT) acquisitions showing a penetrating metallic object (red asterisk) at the level of the right laterocervical region The object had an inferomedial path, traveling immediately tangential to the anteromedial side of the right common carotid artery (red arrow), which remained patent and showed no signs of laceration. The metallic blade was also adjacent to the right lobe of the thyroid and penetrated through the C6-C7 intervertebral space, piercing the C7 vertebral body.

**Figure 3:**
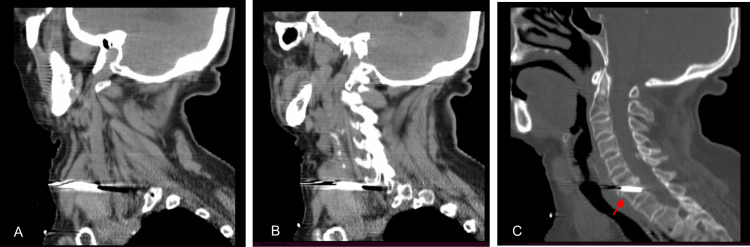
Sagittal enhanced CT reconstructions showing the inferomedial path of the metallic blade piercing the vertebral body of C7 (red arrow)

Arterial blood gas (ABG) analysis revealed the following: pH 7.32, partial pressure of oxygen (pO2) 78 mmHg, oxygen saturation 94%, partial pressure of carbon dioxide (pCO2) 53 mmHg, bicarbonate (HCO3) 27.3 mEq/L and lactates 2.2 mg/dL. The patient was hemodynamically stable so CT scans of the head, neck, and thorax were performed. While the head and thorax CTs offered no relevant findings, the neck CT revealed a metallic blade in situ with a right laterocervical entry wound and an inferomedial oblique path. The blade traveled through the sixth and seventh cervical vertebrae's (C6-C7) intersomatic space and its tip pierced deeply into the body of the seventh cervical vertebra (C7) and the intervertebral disc below. The blade had penetrated between the right lobe of the thyroid and the right common carotid artery, although signs of dissection or laceration were not present (Figures 2A-2C). A hematoma was identified on the right sternocleidomastoid muscle. No intracanal or foraminal involvement was noted (Figures 3A-3C).

Treatment

**Figure 4:**
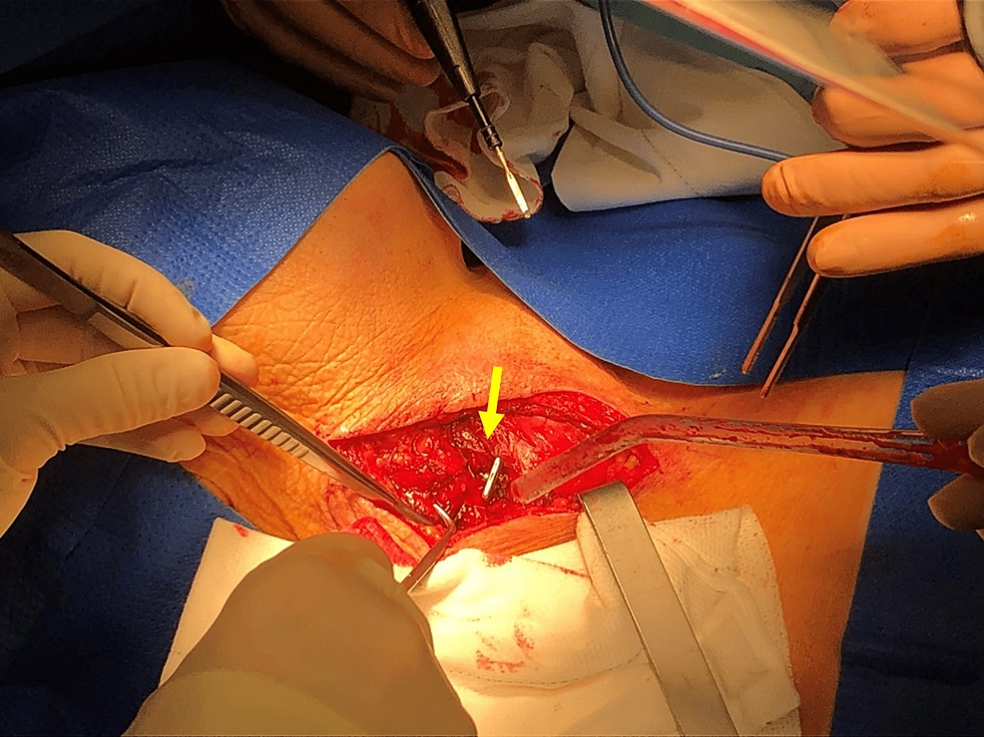
Surgical incision An oblique right cervical incision at the level of the anterior border of the sternocleidomastoid muscle (yellow arrow) was performed. After opening the platysma, surgeons began dissecting the sternocleidomastoid muscle around the blade.

**Figure 5:**
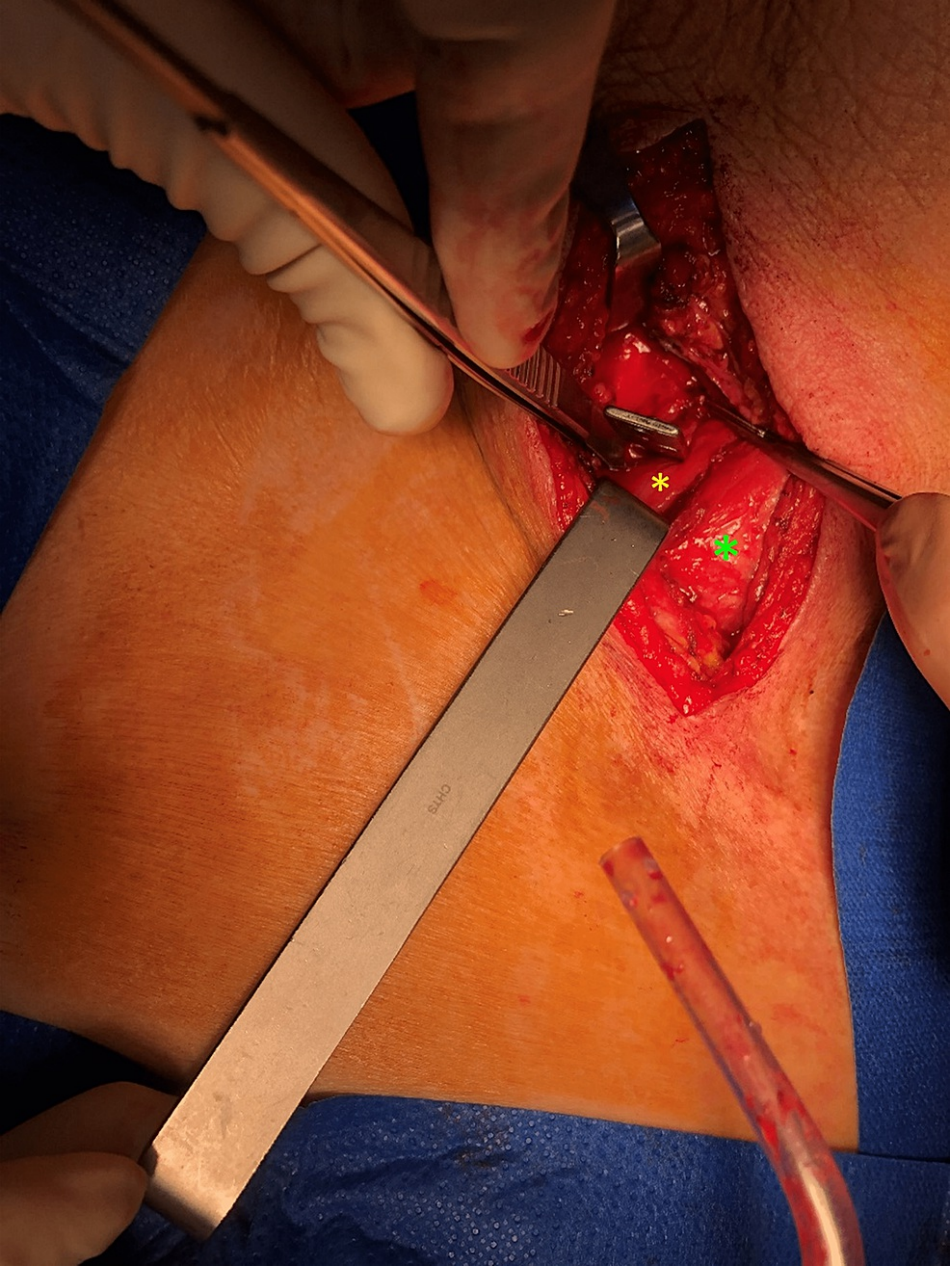
Penetration path of the embedded blade The blade transfixed the sternocleidomastoid muscle (yellow asterisk), penetrating in between the anteromedial side of the right common carotid artery and the right lobe of the thyroid (green asterisk).

After hearing an explanation of the planned procedure, the patient provided consent and underwent a formal neck exploration to achieve controlled extraction of the blade. The close proximity of the blade to the right common carotid artery and the possible need for a complex repair necessitated the presence of two general and two vascular surgeons. In this case, the blood bank was also alerted. In the operating room, the surgeons were careful to ensure posture protection and to avoid any secondary injury to the right common carotid artery due to its close relationship with the blade. The anesthesiologist secured the uninjured airway and placed the patient under general anesthesia, after which surgical exploration began. An oblique right cervical incision at the level of the anterior border of the sternocleidomastoid muscle was performed, extending from the ipsilateral angle of the mandible to the suprasternal notch (Figure 4). After opening the platysma and dissecting the sternocleidomastoid muscle, surgeons noticed that the said muscle was transfixed by the blade. They proceeded to ligate the right superior thyroid artery to release the right lobe of the thyroid from lateral to medial, allowing for greater exposure of the blade’s entire path. Beginning with a penetration path anterior and tangential to the right common carotid artery, the blade then passed posterior to the esophagus and pierced the bodies of the C6 and C7 vertebrae. Neither the common carotid artery nor the esophagus was damaged in the attack (Figure 5).

**Figure 6:**
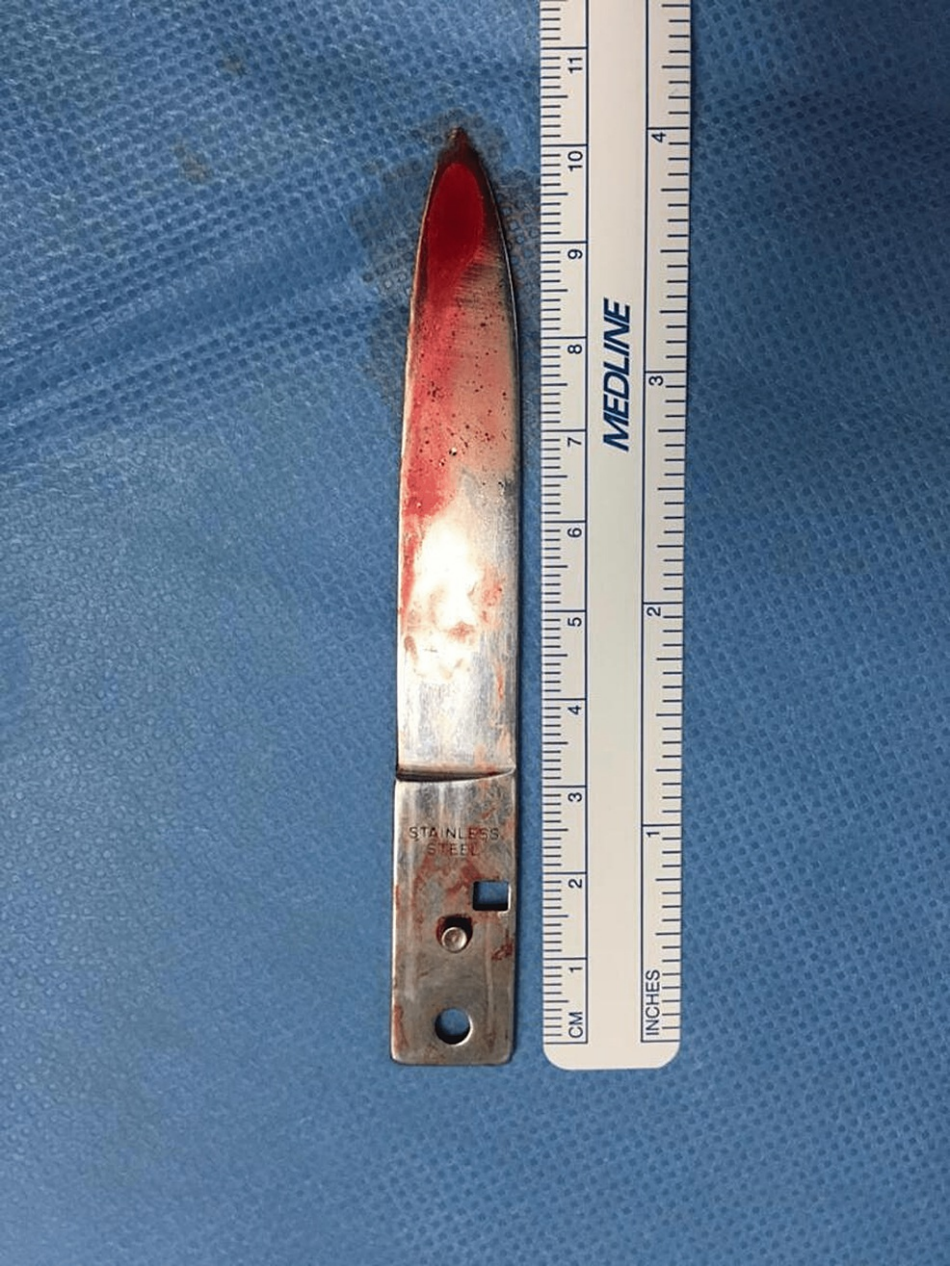
The 10 cm blade extracted

While protecting the common carotid artery, surgeons carefully extracted the blade (Figure 6, Video 1). Hemostasis was secured, and a 28 Fr Blake drain was left in place. The surgeons then closed the platysma and used surgical staples for skin closure.

**Video 1:** Extraction of the impaled blade

Outcome and follow-up

The patient was taken to an intensive care unit where she spent postoperative days one and two, being downgraded to a medical-surgical unit at the end of the second day. On postoperative day four, the 28 Fr Blake drain was safely removed, as drainage volumes had gradually diminished.

A reexamination CT angiography of the neck was performed on postoperative day five; no bleeding complications were found along the blade’s previous path except for some irregularities of the vertebral bodies adjacent to the C6-C7 disc. The patient was discharged the following day. After two years of follow-up, the patient remains free from complications or sequelae.

## Discussion

Monson et al. (1969) divided the neck into three zones: Zone I extends from the sternal notch to the cricoid cartilage, Zone II is between the cricoid cartilage and the angle of the mandible, and Zone III begins at the angle of the mandible and extends proximally to the base of the skull [7]. Monson et al.’s classification has since been used to categorize injuries and guide management decisions.

With unstable patients, the need for immediate surgical exploration is rarely questioned. For the management of stable patients, however, a consensus has yet to be reached. Early algorithms favored mandatory surgery for all Zone II injuries, but the high negative exploration rates and wide availability of CT have led some trauma centers to use a no-zone management algorithm. By choosing this approach, clinical findings and patient stability along with preoperative imaging assessment would support a more selective approach [8,9].

The described case concerns a zone II injury of a patient in stable condition. In this situation, only by performing CT were surgeons able to acknowledge the embedded blade. Prior to the CT examination, no one suspected that the unknown skin prominence noted at palpation was a knife that had broken off in the patient’s neck. Given the patient's stability and undamaged vital structures, surgeons could have considered a conservative approach if it weren’t for the embedded blade.

Some studies from major trauma centers recommend that physical examination alone is adequate for evaluating penetrating neck injuries [10]; however, the authors suggest maintaining a high degree of suspicion and, if the patient’s physiological status is appropriate, performing a CT evaluation. This not only allows surgeons to formulate a plan beforehand but also enables the assembly of the ideal surgical team. In this case, vascular surgeons were present in the operating room and orthopedic surgeons were ready to evaluate cervical spine damage.

Although CT should be the gold standard of preoperative imaging in penetrating neck injuries, it has some limitations that may result in an inability to accurately rule out some aerodigestive tract injuries [11,12]. These are often non-life-threatening injuries that have an insidious course but are associated with a significant risk of morbidity [13]. In this situation, the metallic embedded blade resulted in a scatter artifact that further limited the CT sensitivity.

Since the patient underwent surgical exploration, no other preoperative imaging studies were needed to successfully exclude laryngotracheal or pharyngoesophageal injuries. However, if a conservative approach were to be taken, international guidelines recommend that an endoscopic evaluation of the esophagus or contrast swallow studies should be performed to rule out penetrating esophageal injuries [14]. Besides enabling the diagnosis of esophageal injuries with almost 100% sensitivity, endoscopy also provides for additional assessment of laryngotracheal trauma by allowing the evaluation of the pharynx, larynx, trachea, and bronchi through flexible laryngoscopy or tracheobronchoscopy [15].

## Conclusions

Penetrating neck injuries often look impressive, yet the trauma team must avoid being distracted by such, focusing rather on identifying and treating life-threatening injuries using the primary survey. Although few trauma centers worldwide have a recognizably high volume of penetrating neck injuries, any trauma surgeon can achieve beneficial results by understanding neck anatomy and planning a step-by-step, multi-disciplinary selective approach. If, after CT assessment, surgical exploration is required, two principles must be considered. First, full exposure of the wound must be achieved, as the impaled object should be removed under direct vision. Second, the extraction can only be performed after protecting the surrounding blood vessels and nerves, thus avoiding a secondary injury.
